# Microbial community dynamics in the soil-root continuum are linked with plant species turnover during secondary succession

**DOI:** 10.1093/ismeco/ycaf012

**Published:** 2025-01-29

**Authors:** Weiming Yan, Mengting Maggie Yuan, Shi Wang, Patrick O Sorensen, Tao Wen, Yuting Xu, Honglei Wang, Shuo Jiao, Ji Chen, Zhouping Shangguan, Lei Deng, Ziyan Li, Yangquanwei Zhong

**Affiliations:** State Key Laboratory of Soil Erosion and Dryland Farming on the Loess Plateau, Northwest A&F University, Xinong Road 26, Yangling, Shaanxi 712100, China; Pacific Biosciences Research Center, University of Hawai′i at Mānoa, 2500 Campus Road, Honolulu, HI 96822, United States; Ecology Department, Climate and Ecosystem Sciences Division, Earth and Environmental Sciences Area, Lawrence Berkeley National Laboratory, One Cyclotron Road, Berkeley, CA 94720, United States; Ecology Department, Climate and Ecosystem Sciences Division, Earth and Environmental Sciences Area, Lawrence Berkeley National Laboratory, One Cyclotron Road, Berkeley, CA 94720, United States; Key Laboratory of Organic-based Fertilizers of China and Jiangsu Provincial Key Laboratory for Solid Organic Waste Utilization, Nanjing Agricultural University, Weigang 1, Nanjing, Jiangsu 210095, China; Shenzhen Research Institute of Northwestern Polytechnical University, Sanhang Science & Technology Building, No. 45th, Gaoxin South 9th Road, Nanshan Distict, Shenzhen, Guangdong 518057, China; State Key Laboratory of Soil Erosion and Dryland Farming on the Loess Plateau, Northwest A&F University, Xinong Road 26, Yangling, Shaanxi 712100, China; National Key Laboratory of Crop Improvement for Stress Tolerance and Production, Shaanxi Key Laboratory of Agricultural and Environmental Microbiology, College of Life Sciences, Northwest A&F University, Taicheng Road 3, Yangling, Shaanxi 712100, China; State Key Laboratory of Loess and Quaternary Geology, Institute of Earth Environment, Chinese Academy of Sciences, Yanxiang Road 97, Xi’an, Shaanxi 710061, China; State Key Laboratory of Soil Erosion and Dryland Farming on the Loess Plateau, Northwest A&F University, Xinong Road 26, Yangling, Shaanxi 712100, China; State Key Laboratory of Soil Erosion and Dryland Farming on the Loess Plateau, Northwest A&F University, Xinong Road 26, Yangling, Shaanxi 712100, China; College of Resources and Environment, Northwest A&F University, Taicheng Road 3, Yangling, Shaanxi 712100, China; Shenzhen Research Institute of Northwestern Polytechnical University, Sanhang Science & Technology Building, No. 45th, Gaoxin South 9th Road, Nanshan Distict, Shenzhen, Guangdong 518057, China

**Keywords:** endosphere, rhizosphere, microbial recruitment, plant turnover, succession

## Abstract

Grazing exclusion and land abandonment are commonly adopted to restore degraded ecosystems in semiarid and arid regions worldwide. However, the temporal variation in the soil- versus root-associated microbiome over plant species turnover during secondary succession has rarely been quantified. Using the chronosequence restored from fenced grassland and abandoned farmlands on the Loess Plateau of China, we characterized the dynamics of the soil- and root-associated microbiome of host plant with different dominance statuses during secondary succession from 0 to 40 years. Our results revealed that the root microhabitat, the host plant and their interactions were the main contributors to the bacterial community shift (R^2^ = 15.5%, 8.1%, and 22.3%, respectively), and plant interspecies replacement had a greater effect on the shift in the root-associated microbial community than intraspecies replacement did during succession. The root-associated bacterial community of pioneer plants was particularly responsive to succession, especially the endosphere community. Endosphere microbial diversity was positively correlated with host plant coverage change, and the diversity and abundance of taxon recruitment into the endosphere of pioneer plants from the surrounding environment decreased as succession progressed. The community assembly processes also indicated that the endosphere microbiota are strongly selected in younger host plants, whereas stochastic processes dominate in aged host plants. Our study provides evidence of the unique response of the root-associated microbiome to the replacement of plant species during secondary succession, and the function of endosphere microbes should be considered when studying plant–microbe feedback.

## Graphical Abstract

**Graphical Abstract:**
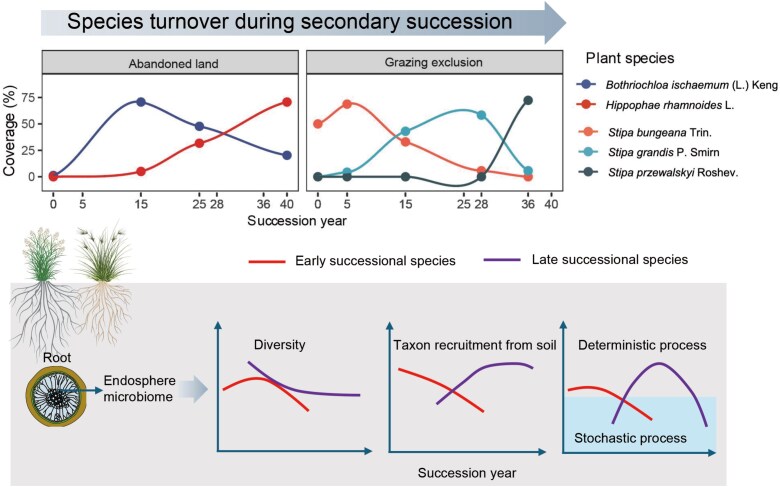

## Introduction

Earth’s drylands are experiencing degradation as a result of unsustainable land use practices, such as overgrazing, extensive cropping, and deforestation [1, 2], which may be exacerbated under climate change [3]. Thus, “nature-based” solutions, such as grazing exclusion and land abandonment have been implemented globally [4, 5] to foster secondary succession of natural plant communities, with co-benefits including improved soil structure and soil fertility [6, 7]. The soil microbiome, which includes both microbes living in bulk soil- and root-associated microbes, plays an important role in the sustainable development of plant communities after environmental disturbances [8]. As such, understanding the ecological principles governing shifts in the soil- and root-associated microbiome communities of host plants with different dominance statuses could offer valuable insights into plant replacement dynamics during secondary succession

Diverse communities of microorganisms colonize the interior and exterior of plant roots, collectively known as root-associated microbiota [9, 10], which have been found to confer numerous benefits to plants including growth promotion as well as biotic and abiotic stress resistance [11, 12]. The composition of the root-associated microbiota depends on the soil microbial seed bank as well as selection by the host plant [13–15]. The growth, senescence and turnover of host plants over time during succession may alter root-associated microbial community composition and function [16], which in turn influences plant community stability. Previous studies have shown that the root-associated microbiome changes across plant growth stages [10, 15, 17, 18]. For example, the root-associated microbiota often has a high turnover rate of community composition during the vegetative phase and then stabilizes compositionally throughout the remainder of the lifespan of annual plants [10]. We therefore might assume that the root microbiome is highly related to the host plant growth status in the community during succession. However, the role of root selection in shaping the structure of the root-associated microbiota during host plant turnover caused by succession is broadly unknown.

Secondary succession after disturbance most often begins with pioneer species colonizing harsh and oligotrophic environments, and over time plant species with more competitive traits displace pioneering species, which often improves the soil environment [19]. As succession progresses, the compositions of the plant and soil microbes evolve, leading to a more mature and stable ecosystem [5, 20]. Management strategies, climates, land use histories and plant–soil feedback driven by changes in the microbiome may be the crucial factors influencing the dynamics of vegetation successional patterns [21, 22]. At some sites, plant species turnover happens within the same functional group (e.g. grass) or plant species may shift from one functional group to another (e.g. grass to shrub) at the other sites. Given that soil- and root-associated microbiota play an important role in plant growth and replacement during succession [10, 23, 24], understanding the composition and assembly dynamics of root-associated microbiota and their recruitment process in different host plant species during secondary succession is crucial.

In this study, we investigated the microbial community diversity, composition, assembly and recruitment process along the soil–root continuum of host plants with different dominance statuses during secondary succession ranging from 0 to 40 years, following grazing exclusion and farmland abandonment. We hypothesized that (i) root-associated microbial diversity and composition vary across different host plants and succession types, which is related to host plant replacement; (ii) the root-associated microbial community of pioneer host plants might be more responsive to succession than that of late-successional plants; and (iii) microbial communities in the endosphere would be the microbiome compartment most sensitive to plant replacement during secondary succession.

## Materials and methods

### Study sites

**Figure 1:**
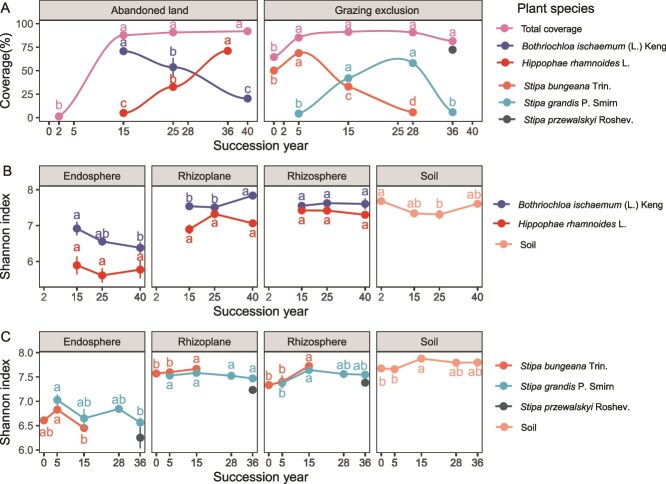
Plant species coverage and microbial diversity dynamics during two types of secondary succession. (A) Coverages of the total community and specific plant species dynamics during the succession process. *B. ischaemum* (L.) Keng is the pioneer species, and the short shrub *H. rhamnoides* L. is a later species at land abandonment site. *S. bungeana* Trin. is the pioneer species, and *Stipa grandis* P. Smirn and *S. przewalski* Roshev. are the later species at the grazing exclusion site. Microbial Shannon indices of soil and root-associated microbes in dominant and nondominant plant species during with succession following land abandonment (B) and grazing exclusion (C). The dots indicate the means, and the error bars represent the standard error. Lowercase letters indicate significant differences among succession years, which were determined via one-way ANOVA and post hoc comparisons via Tukey’s multiple-range test.

Two sites were selected in the semiarid area of the Loess Plateau, China, to represent secondary succession stages ranging from 0–40 years after restoration started (Fig. S1A). The first site is located at the Lianjiabian Forest Farm in the Ziwuling forest region in Gansu Province, China (35°03′-36°37′N,108°10′-109°18′E, 1211–1453 m a.s.l.), where the annual temperature is 10°C and the annual precipitation is 587 mm. At this site, the restoration and secondary succession of vegetation has naturally occurred on abandoned farmland (maize and foxtail millet were the main rotational crops) in this region for ~170 years [16]. In this study, we operationally defined and studied three vegetation recovery stages: (i) the grassland stage (~15 years of succession, AL15), in which *Bothriochloa ischaemum* (L.) Keng is the dominant species and the short shrub *Hippophae rhamnoides* L. is distributed sporadically; (ii) the latter grassland stage (~25 years of succession, AL25), in which grass coverage decreases and shrub coverage increases; and (iii) the early shrubland stage (~40 years of succession, AL40), in which *H. rhamnoides* dominates the community and *B. ischaemum* is distributed sporadically. In addition, we chose newly abandoned farmland as the control treatment (2 years of succession, AL2), in which *H. rhamnoides* and *B. ischaemum* had not emerged (Fig. 1A).

The second site is located at the Yunwushan National Natural Grassland Reserve (106°21′–106°27′ E, 36°10′–36°17′ N, 1800–2100 m a.s.l), with an average annual precipitation of 425 mm and an average annual temperature of 7°C. This area has been gradually restored from degradation caused by overgrazing by implementing grazing exclusion since 1982. At this site, four grassland sites along the chronosequence allowing natural restoration since 1982, 1990, 2003, and 2013 were selected, corresponding to grazing exclusion for 36 years (GE36), 28 years (GE28), 15 years (GE15), 5 years (GE5), and one grazed site (4 sheep/ha), which were chosen as the control treatment (GE0) [20]. After grazing exclusion, the plant community is dominated by *Stipa* spp., with a notable replacement of *Stipa bungeana* Trin. by *Stipa grandis* P. Smirn. and *Stipa przewalski* Roshev. as the duration of grazing exclusion increases. The detailed plant species replacement information for each site is shown in Table S1.

### Bulk soil sampling and root-associated (rhizosphere, rhizoplane, and endosphere) fraction collection

Bulk soil and root samples from nine selected field sites were collected in August 2018. At each sampling site, we laid a 100 m transect and established four 3 × 3 m quadrats at 20 m intervals (5 m × 5 m for shrubland stage). Ten soil samples were randomly collected and combined from the 0–20 cm soil layer in each replicate plot at each stage using a soil auger (5 cm inner diameter), resulting in 36 soil samples in total. All the soil samples were sieved through a 2-mm screen to remove roots and other debris. A portion of each soil sample was collected in a 10 ml centrifuge tube, which was immediately frozen and stored in liquid nitrogen and transferred to the laboratory. The tubes were maintained at −80°C until soil deoxyribonucleic acid (DNA) extraction.

The roots of the five target species were collected, and excess soil was manually shaken off, leaving ~1 mm of soil attached to each root. The roots were cut into ~5 cm segments using sterile scissors, and stored in a 50 ml tube in liquid nitrogen, and transported to the laboratory for the isolation of the rhizosphere, rhizoplane, and endosphere microbiomes [25]. Briefly, rhizosphere soil from the roots was directly separated by placing them in a sterile flask with 50 ml of sterile phosphate-buffered saline (PBS) solution, and the roots were stirred vigorously with sterile forceps. The resulting soil suspension was collected with a 50 ml Falcon tube and stored at −80°C until DNA extraction, which was designated as the rhizosphere soil. For the collection of the rhizoplane microbiome, the roots were cleaned and placed in a Falcon tube with 15 ml of PBS and were sonicated for 30 seconds with 50–60 Hz, after which the liquid PBS fraction was collected as the rhizoplane soil. Finally, the roots were surface sterilized with 5% (w/v) NaClO solution for 1 min, and then were sonicated and shaken again with 25 ml of PBS, which was repeated two times. The cleaned roots were subsequently cut into 2 mm segments and homogenized in liquid nitrogen for DNA extraction (Fig. S1B). In total, 4 parts of the soil- and root-associated microbial communities were collected with different host plant species in each stage at each site, resulting in 204 DNA samples, including 36 soil samples from two sites, 56 from the endosphere, 56 from the rhizosphere and 56 from the rhizoplane for five plant species.

### Deoxyribonucleic acid extraction, ion S5 XL sequencing, and data processing

Microbial DNA of rhizoplane, rhizosphere, and bulk soil was extracted from 0.5 g soil of each fraction. The endosphere fraction was extracted from 1 g of root samples after pre-homogenized before the DNA extraction by bead beating for 1 minute [10]. All microbial DNA extraction was using the E.Z.N.A DNA kit (Omega Biotek, Norcross, GA, USA) according to the manufacturer’s protocol. The V4 region of the bacterial and archaeal 16S rRNA gene was PCR amplified (95°C for 2 min followed by 27 cycles at 95°C for 30 s, 55°C for 30 s, and 72°C for 45 s, with a final extension at 72°C for 10 min) using the primers 515F (5′-barcode-GTGYCAGCMGCCGCGGTAA-3′) and 806R (5’-GGACTACNVGGGTWTCTAAT-3′) [26]. All PCRs were performed using Phusion® High-Fidelity PCR Master Mix (New England Biolabs, Hertfordshire, UK). Sequencing libraries were generated using the Ion Plus Fragment Library Kit 48 rxns (Thermo Fisher Scientific, Waltham, MA, USA) following the manufacturer’s recommendations. Library quality was assessed using a Qubit@ 2.0 fluorometer (Thermo Fisher Scientific, Waltham, MA, USA) and then sequenced on an Ion S5™ XL platform.

Reads from 16S-V4 sequencing were analyzed using QIIME 2 (v2018.4) [27]. Reads from 16S-V4 were trimmed where the average quality score dropped <25 and were dereplicated using DADA2 as implemented in QIIME 2 with a paired-end setting (including quality control, trimming, pair-joining, and chimera removal), resulting in 91.96% of the reads being retained. The 16S-V4 representative amplicon sequence variants (ASVs) were assigned taxonomy using the SILVA 128 database [28] and the naïve Bayes classifier in QIIME 2 to produce taxonomy tables. The representative sequences, taxonomies, and count tables from the bacterial reads were merged in QIIME 2. Phylogenetic trees were built in QIIME 2 using MAFFT alignment in QIIME 2 and the FastTree algorithm [29]. On average, 79 352 high-quality 16S rRNA gene sequences were obtained per sample, and 29 818 bacterial and archaeal ASVs were detected. To avoid chloroplast contamination, we filtered the chloroplast reads for the following analysis (Table S2). All samples were normalized to equal sampling depths for subsequent data analysis. The bacterial 16S rRNA gene sequencing data were uploaded to the NCBI SRA database under accession number PRJNA1018273.

### Plant coverage investigation and soil physicochemical analysis

The coverage of the target plant in the grassland was determined using a 1 × 1 m grid with 100 crosshairs (each grid cell was 10 × 10 cm^2^). The shrub crown width was measured to calculate the shrub coverage within a 5 × 5 m area [30]. The soil pH was determined via a glass electrode with a 1:2.5 soil:water ratio by mass following established protocols [31]. The soil organic carbon content was quantified via oxidation with potassium dichromate in a heated oil bath [32]. Total nitrogen was assayed via the Kjeldahl method [33]. All the soil physicochemical analyses were repeated and averaged for each replicate (Table S1).

### Calculations and statistical analyses

All the statistical analyses were performed via the R software package (version 4.0.3) [34]. The bacterial Shannon index and abundance-based coverage estimator (ACE) were calculated with the “diversity” and “estimatedR” functions in the R package “vegan” (version 2.5.7). Analysis of variance (ANOVA) was used to detect the differences in bacterial diversity among different successional stages, Post hoc comparisons were conducted via Tukey’s honestly significant difference (HSD) test, and the significance level was set at *P* < 0.05. Nonmetric multidimensional scaling (NMDS) with the Bray–Curtis distance matrix was implemented with the “metaMDS” function in R package “vegan” (version 2.5.7). Permutational-based ANOVA (PERMANOVA, permutations = 999) was used to test the significance of each subset group via the “adonis” function in R package “vegan” (version 2.5.7). The microbial community turnover rate was evaluated via the change in the bacterial community composition similarity (Bray–Curtis distance) with successional years using linear regression.

Beta regression using the “safe_betareg” function in “betareg” package (version 3.1.4) [35] was performed to model increasing or decreasing relative abundances of individual phyla between the various root-compartments in all succession stages [10]. We assigned each compartment a value relative to its spatial position: the bulk soil was position 1, the rhizosphere was position 2, the rhizoplane was position 3, and the endosphere was position 4. For the compartment model, we combined all successional stages for each plant. For the successional model, we detected changes in the phyla in each compartment during succession.

The common and core taxa of the four root-compartments, which we defined as ASVs present in all four microhabitats in each plant succession stage, were selected in different plant species to infer root recruitment processes during succession. Full random forest models were generated and analyzed using the “randomForest” package (version 4.6.14) [36] to select the sets of ASVs found in the endosphere and distinguish the samples by successional year (succession-discrete ASVs) for each plant species. Variation in phylogenetic diversity and stochastic versus deterministic microbial community assembly processes were quantified using null model-based phylogenetic β diversity analysis to test for the various community assembly processes via the “picante” package (version 1.8.2) [37].

## Results

### Diversity and compositional shifts of soil- and root-associated microbiota during succession

The bacterial α diversity varied among the soil and root-compartments and exhibited distinct dynamic patterns among the different host plants at both two successional sites. In the bulk soil, bacterial Shannon diversity and ACE richness decreased but then increased after 25 years at the AL site (Figs 1B and S2A). In contrast, the α diversity increased for 15 years following grazing exclusion, after which it began to decrease at the GE site (Figs 1C and S2B). The rhizosphere and rhizoplane microbial diversity of the pioneer species *S. bungeana* increased with successional year at the GE site. Interestingly, the endosphere α-diversity decreased from 6.8 to 6.3 in *B. ischaemum*, from 6.8 to 6.4 in *S. bungeana* and from 7.0 to 6.5 in *S. grandis* (Figs 1B and C and S3), indicating positive relationships with their host plant coverage change (Fig. S4).

**Figure 2:**
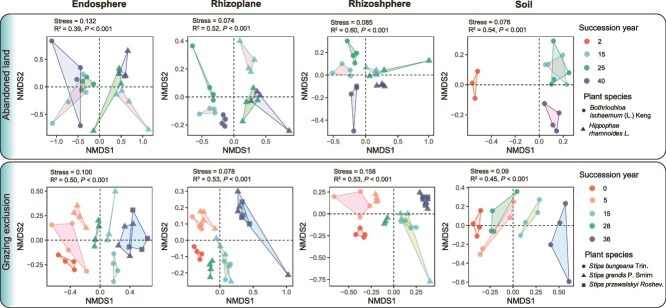
NMDS analysis of the soil and root-associated microbiota communities of different host plants on the basis of the Bray–Curtis distances of land abandonment (A) and grazing exclusion (B) successional sites. P and R^2^ were calculated via PERMANOVA via Adonis.

The root microhabitat, host plant, succession type, duration of restoration and their interactions significantly affected the microbial composition; microhabitats, host plants and their interactions were the main contributors to the bacterial community shift (adonis R^2^ = 15.5%, 8.1%, and 22.3%, respectively, Table S3). At the AL site (interspecies succession), the microbial communities were influenced predominantly by microhabitats and host plants (Fig. 2A, Table S4). At the GE site (intraspecies succession), the microbial communities were affected by the succession year, the host plant and their interaction. Notably, the succession year had a more significant impact than the host plant; thus, the samples displayed a spatial pattern of divergence along the first principal coordinate with successional years in each microhabitat (Fig. 2B).

The community turnover rate of the root-associated microbiota (evaluated as the change in community similarity with time) exhibited different trends among different host plants throughout the succession (Fig. S5). The endosphere community similarity decreased with successional year for the three herb species. At the GE site, the community similarity of the root-associated microbiota decreased over time after grazing exclusion increased, and the similarity of the microbiome associated with the pioneer species *S. bungeana* declined faster than that associated with the later colonizing plant species.

### Relative abundance of specific phyla that shifted in each compartment during succession

**Figure 3:**
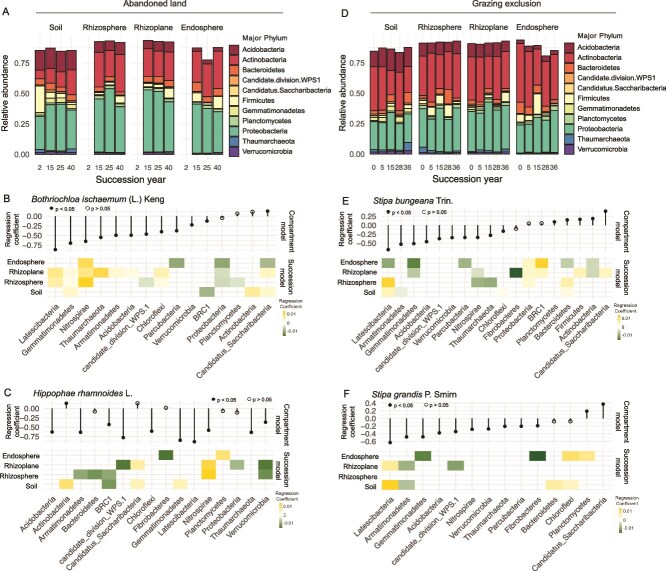
Responding phyla in the soil and root-associated microbiota during succession. Bar plots of the abundances of the top 11 phyla in the soil and root-associated microbiota during succession at land abandonment site (A) and grazing exclusion site (D). Each bar represents 1 successional stage in a different compartment. (B, C, E, F) Upper panel: beta regression coefficient estimates for microbial phyla whose relative abundance either increased (>0) or decreased (<0) from the outside of the root to the inside of the root. Lower panel: Beta regression coefficient estimates for microbial phyla whose relative abundance increased (>0) or decreased (<0) over the course of succession in each compartment. *B. ischaemum* (L.) Keng (B) and *H. rhamnoides* L. (C) in succession following land abandonment and *S. bungeana* Trin. (E) and *S. grandis* P. Smirn (F) in succession following grazing exclusion.

The relative abundance of the dominant microbial phyla differed among root microhabitat during succession at the two sites. Proteobacteria (average abundance 34.9%) and Actinobacteria (average abundance 29.4%) were the two most abundant phyla in each compartment at both sites (Fig. 3A and D). By identifying specific phyla that significantly differed from the exterior to the interior of the root using beta regression for each dominant species, we identified a greater number of root-depleted phyla compared to root-enriched phyla (Fig. 3B, C, E, and F, upper panel). Interestingly, although the two plant species grew under the same soil conditions, the relative abundances of certain root compartment-responding phyla were different (Fig. 3B, C, E, and F, lower panel). Microbes in the endosphere were primarily recruited from the rhizoplane plus rhizosphere, but changes in phylum composition within the endosphere did not mirror changes in the rhizoplane or rhizosphere. For example, the abundance of most phyla within the endosphere community decreased in abundance as the length of succession increased, but the abundance of only a few phyla increased as succession progressed. Furthermore, Nitrospirae increased and Proteobacteria decreased in all the root compartments throughout succession in *B. ischaemum* at the AL site. However, there are more taxa changed in pioneer plants with succession at the GE site, e.g. Proteobacteria and BRC1 demonstrated a consistent increase in the endosphere and rhizoplane of *S. bungeana*, while Latescibacteria, Gemmatimonadetes, Parcubacteria, Bacteroidetes, and Actinobacteria in the endosphere significantly declined (Fig. 3B and E).

### Common taxa in the four microhabitats and discriminant biota differed among host plants during succession

The results revealed that the bacterial community similarity between the endosphere and rhizoplane of pioneer plants decreased with successional years at both sites (Fig. S6), implying that the recruitment potential of the roots from the exterior to the interior decreased with secondary succession. To test these results, we identified the common ASVs in four root compartments for each plant species at each successional stage. The results revealed that the abundance and diversity of common ASVs declined in the endosphere of the pioneer plants with succession (Fig. S7A and C), which was not observed in the later colonizing plants during later successional process (Fig. S7B and D).

**Figure 4:**
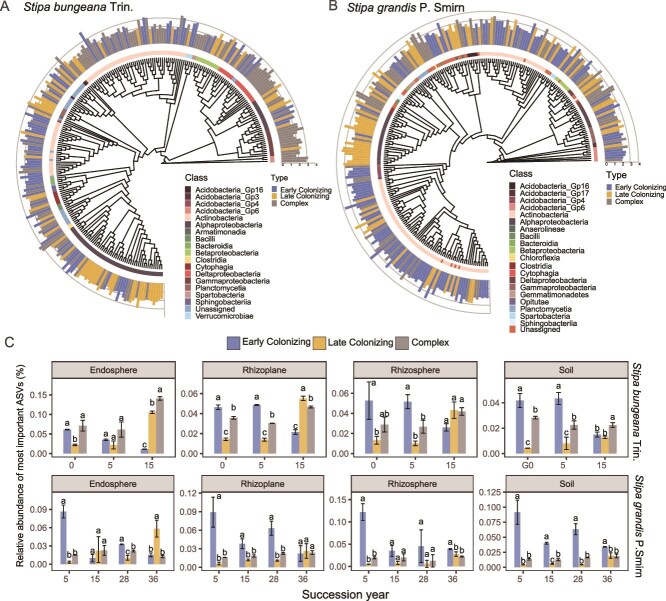
Discriminant ASVs of the soil and root-associated microbes during succession. Neighbor-joining tree of discriminant ASVs showing significant changes in the endosphere compared with the other compartments in early successional *S. bungeana* Trin. (A) and later successional *Stipa grandis* P. Smirn (B). The bars moving away from the tree represent early (significantly decreasing over the course of the succession), late (significantly increasing over the course of the succession) and complex (not significantly different than 0 over the course of the succession) colonizing discriminant ASVs in the endosphere over the course of the succession, respectively. The bar height represents the importance value of each ASV (mean decrease accuracy). (C) Abundance profiles for the successional discriminant ASVs in the endosphere over the course of the successional years at the grazing exclusion site. ASVs are colored by their classification as decreasing, increasing, or neutral colonizers throughout the succession.

In addition, we identified the sets of endosphere ASVs that could predict succession time via random forest classification, and our results revealed better performance at the GE site than at the AL site (low error rate, Fig. S8; AL site results shown in Fig. S9). We found that these important ASVs were more abundant in the endosphere than in the surrounding environment (Fig. 4C). The relative abundances of early colonizer taxa gradually decreased in accordance with their abundances in the rhizosphere, rhizoplane and soil. In contrast, later colonizers presented much greater abundances in the endosphere of the host plant in the last successional stage (15 years for *S. bungeana* and 36 years for *S. grandis*; Fig. 4C).

### The microbial community assembly process showed different trends in species turnover during succession

**Figure 5:**
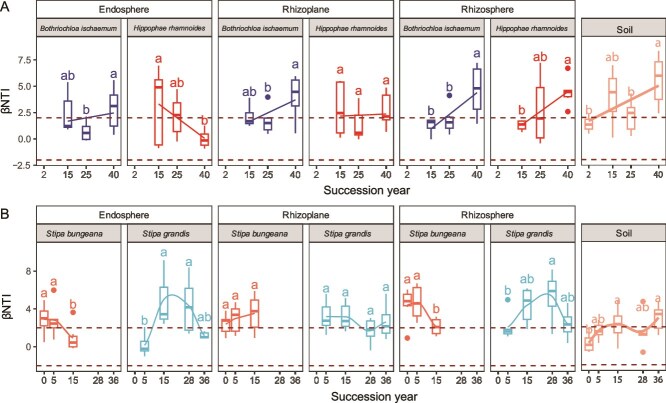
The between-community NTI (βNTI) of the soil and root-associated microbial communities along with succession at land abandonment site (A) and grazing exclusion site (B). The horizontal bars within the box plots represent medians. The tops and bottoms of the boxes represent the 75th and 25th percentiles, respectively. Lowercase letters indicate significant differences among succession years, which were determined via one-way ANOVA and post hoc comparisons using Tukey’s multiple-range test.

The null model-based analysis revealed that stochastic community assembly processes (|βNTI| < 2) dominated microbial community assembly in the early stage of succession (<5 years) at both sites, whereas deterministic processes (|βNTI| > 2) contributed more to soil microbial community assembly at later successional stage (Fig. 5). βNTI declined for the endosphere community of *H. rhamnoides* at the AL site and deterministic processes dominated patterns of assembly for the root-associated microbiota in *B. ischaemum* in the late-successional stage (Fig. 5A). The endosphere of the pioneer grass (*S. bungeana*) was initially dominated by deterministic processes but later shifted toward stochastic processes as time since grazing exclusion increased (Fig. 5B). In contrast, the microbial community assembly of the later colonizing plant (*S. grandis*) was initially dominated by stochastic processes and then shifted to deterministic processes with succession progressed, and again shifted back to be determined largely by stochastic processes in the later successional stage.

## Discussion

### Turnover of host plants impacts root-associated microbial community diversity and composition during succession

The α diversity of root-associated microbes exhibited distinct spatiotemporal variations in relation to changes in host plants during the two successional patterns (Figs 1, S2, and S3). The positive correlation between endosphere microbial diversity and plant coverage of pioneer species suggests that a more diverse endosphere community may benefit plant colonization at the early successional stage [10, 38]. Moreover, senescence and degradation of host plants generally lead to decreased microbial diversity in the endosphere. In contrast, the rhizoplane and rhizosphere microbial diversity displayed a positive relationship with succession year, indicating that microbes in the rhizosphere and rhizoplane of host plants are more strongly impacted by soil nutrients than those in the endosphere [25].

Furthermore, we detected a greater effect of plant interspecies succession (AL site) than intraspecific succession (GE site) on root-associated microbial communities (Fig. 2). Host plants are the most important factor driving root-associated microbial communities shifts at the AL site, likely because of the differences in adaptive physiological traits that confer different life history strategies to the two dominant host plants [39, 40]. For example, *B. ischaemum* is a C4 grass, and *H. rhamnoides* is a shrub that has been observed to build associations with *Frankia actinomycetes* to enable symbiotic nitrogen fixation [41], which could improve soil fertility during succession. At the GE site, the soil- and root-associated microbial communities were influenced more strongly by the year of succession since grazing exclusion than by the host plant. One explanation for this phenomenon could be related to the change in soil nutrients with succession (Table S5). In addition, the microbial communities in the rhizoplane and rhizosphere of both host plants in the same year at the GE sites were highly similar (Fig. 2B), possibly because the comparable root characteristics and exudates produced by these functionally similar plants (both are *Stipa* genus) did not have strong effects on shaping these microbes [15, 25, 42, 43]. The greater impact of the host plant on the endosphere communities at the GE site indicated that the endosphere community was not only affected by the species pool from the surrounding environment but also sensitive to the host plant [44]. The above results highlight the importance of both soil nutrients and host plants in shaping soil- and root-associated microbiomes. High interspecies diversity might lead to greater niche differentiation, which could increase the complexity and resilience of microbial communities [45]. These results carry important implications for ecological restoration that the interspecies vegetation community might have a more divergent microbial community.

### Root-associated microbes of pioneer species plant are particularly responsive to succession

The turnover rate of root-associated microbial communities differed chronologically as the dominant plant species changed (Fig. S5), supporting previous research reporting that plant growth affects the root-associated microbiota [10, 46–48]. We observed a greater turnover rate of root-associated microbiota in pioneer plant species (*B. ischaemum* and *S. bungeana*) than in later colonizing plants (Fig. S5). One explanation for this phenomenon is likely that pioneer plants often have shorter life cycles, which may lead to fast turnover of their root-associated microbes [49]. In addition, pioneer plants typically inhabit disturbed or harsh environments with low competition, the fast turnover of microbes could facilitate plant immune responses and the acquisition of nutrients and water [50]. These results could indicate that the root-associated microbiota in pioneer plants is more sensitive to succession than the microbiota associated with later colonizing plant species [10]. Studies have shown that early pioneer plants often experience strong negative plant–soil feedback from certain soil microbes, which promotes their replacement by later-successional species with improvements in the edaphic environment and greater resistance to biotic stress [23, 51, 52]. For example, negative plant–soil feedback leads to the accumulation of plant pathogens in early colonizing plants [53], and the growth of early colonizing plants in soils conditioned by late-successional species could be severely hindered [23, 54, 55]. The presence of nitrogen-fixing bacteria (e.g. Nitrospirae increased with succession at the AL site; Fig. 3A) and root nodules can also lead to positive plant–soil feedback, supporting the colonization of later plant species [24]. The faster changes in the root-associated community in early pioneer plants suggest that this community is closely related to host plant growth, as they mainly obtain the energy and substrate from the roots in the soil with insufficient nutrients.

Previous studies have focused mainly on microbial turnover in different plant growth stages of annual plants [46–48]. Our study utilized a 0 to 40 year chronosequence to understand the dynamics of the root-associated microbiota in host plants with different dominance statuses during succession. Our results support our hypothesis that the root-associated community is particularly responsive to succession, especially for pioneer plants. The findings from this study highlight the dynamic relationships between host plant growth and the root-associated microbiota, which should be considered when understanding the turnover of both the root-associated community and host plants during secondary succession.

### Microbial taxa recruitment into the endosphere of pioneer species plants decreased as succession progressed

The endosphere microbiome is selectively filtered from the surrounding environment (rhizoplane, rhizosphere and bulk soil) [25, 56, 57]. In this study, the abundance of most major phyla decreased from the bulk soil to the endosphere (Fig. 3B, C, E, and F, upper panel), confirming the strong selective role of roots in the endosphere compartment during longer succession [10, 17, 57, 58]. The taxa enriched or depleted in the endosphere during succession are affected by host plants due to their different functional traits, such as nutrient uptake strategies and root architecture [59]. Rapidly changes in endosphere community composition and a reduction in similarity between the endosphere and rhizoplane communities in pioneer plants suggest that the taxon recruitment into the endosphere of early pioneer plants decreased along with secondary succession (Figs S6 and S7). In general, when plants settle in a disturbed environment, communities of early colonizing microbes begin to establish inside and near the roots, which would be displaced by later colonizing microbes with the growth and demise of plants [10]. Similarly, perennial herbs growth is often affected by the plant immune system [60], which may influence the recruitment ability of the endosphere in aged host plants during succession.

By employing the random forest classification approach, we found that the abundance of early colonizing microbes in the endosphere gradually decreased as their source bank (rhizoplane, rhizosphere and soil) abundance declined during succession at the GE sites. The later colonizing microbes presented opposite trends and were enriched in the endosphere at the late-successional stage (Fig. 4C). These results indicate that the late-emerging microbiota were more strongly selected by host plants in later succession stages [23], which is consistent with the findings of previous study [10]. These results imply that specific ASVs in the endosphere could be indicators of host plant succession. Some studies have shown that these later colonizing taxa may be harmful microbes that make plants susceptible to disease or replacement when they are in unfavorable environments [61]. However, further studies are needed to reveal the function of how these late-emerging microbiota are related to plant degradation. Overall, our study highlights the dynamic changes in the endosphere microbial community in pioneer plants from flourishing to aging over long-term succession.

### The stochastic and deterministic process of root-associated microbial community assembly shifts during succession

The microbial community assembly process has been previously investigated during succession [62, 63], and our results support the observations made during salt marsh succession [62], in which the microbial composition of bulk soil was initially governed by stochasticity at the early succession stage (<15 years). However, the deterministic processes indicative of adaptive selection progressively dominated microbial community assembly along with succession at both sites (Fig. 5), which may also be associated with improvements of the edaphic environment [64].

Microbial accumulation within root-compartments varies among host plants and results in corresponding changes in the community assembly process. Previous studies revealed different dominant assembly processes of the microbial community in the endosphere and rhizosphere [25, 65]. Our results also revealed that the relative importance of stochastic and deterministic processes varied among root-compartments in different host plants during succession, and the rhizoplane and rhizosphere communities were predominantly influenced by deterministic processes in the later successional stage at the AL site and at all stages at the GE sites (Fig. 5). Similarly, the community assembly processes of endosphere communities in *S. grandis*, *S. bungeana* and *H. rhamnoides* are predominantly governed by deterministic processes, indicating the strong selective function of roots for the specific functional microbial taxa from the surrounding environment to support plant growth [66]. However, stochastic processes play a more important role in aged host plants at the later successional stage, supporting the concept that stochastic processes can drive random phylogenetic turnover of endosphere microbial communities when plants lose competitive edges [67]. These results imply that root selection plays a crucial role in shaping microbial communities, which in turn help the host plants adapt to the environment and affect ecosystem functions.

Our study provides valuable insights into the temporal dynamics of soil- and root-associated microbiomes during secondary succession with different types of host plant replacement. However, several limitations must be acknowledged. First, the chronosequence approach relies on space for time substitutions, which might introduce some uncertainties due to the environmental factors at each site. Additionally, the functions of the identified bacterial communities, as well as root-associated fungal communities should be clarified in future, to better understand their roles in nutrient cycling and plant growth support along secondary succession.

## Conclusion

In this study, we have shown here that the variation of soil and root-associated microbial communities depends on the succession type, year and host plant and that the influence of host plants under interspecies succession is greater than that under intraspecific succession. Our results indicate that the root-associated community is particularly responsive to changes in host plants, especially the endosphere community in pioneer plants. The diversity and abundance of taxa recruitment in the endosphere decreased as succession progressed of pioneer plants. The community assembly processes also indicated that the endosphere microbiota is strongly selected in younger host plants, whereas stochastic processes dominate in aged host plants. Overall, our study provides evidence of the unique response of root-associated microbiota along with host plant turnover, and the function of endosphere microbes should be considered when studying plant–microbe feedback during secondary succession.

## Supplementary Material

Supplementary_information_ycaf012

## Data Availability

All information is included in this published article and its supplementary information files. The 16S sequence data were submitted to the NCBI SRA under accession number PRJNA1018273. All codes used for this study are available online at https://doi.org/10.6084/m9.figshare.28238126.v1.
