# Supplementary material for: Microbial community dynamics in the soil-root continuum are linked with plant species turnover during secondary succession
Source: ISME Commun. 2025 Jan 29;5(1):ycaf012. doi: 10.1093/ismeco/ycaf012 (PMC11932647; doi:10.1093/ismeco/ycaf012)
Supplement: Supplementary_information_ycaf012 [file supplementary_information_ycaf012.pdf]

Supporting Information

Article title: Microbial community dynamics in the soil-root continuum are linked with plant species turnover during secondary succession

Authors' names: Weiming Yan[1], Mengting Maggie Yuan[2], Shi Wang[3], Patrick O. Sorensen[3], Tao Wen[4], Yuting Xu[5], Honglei Wang[1], Shuo Jiao[6], Ji Chen[7], Zhouping Shangguan[1], Lei Deng[1], Ziyan Li[8*], Yangquanwei Zhong[5*]

The following Supporting Information is available for this article:

Figure S1 Map of study sites and methods of sampling and collection of the soil and root-associated microbes

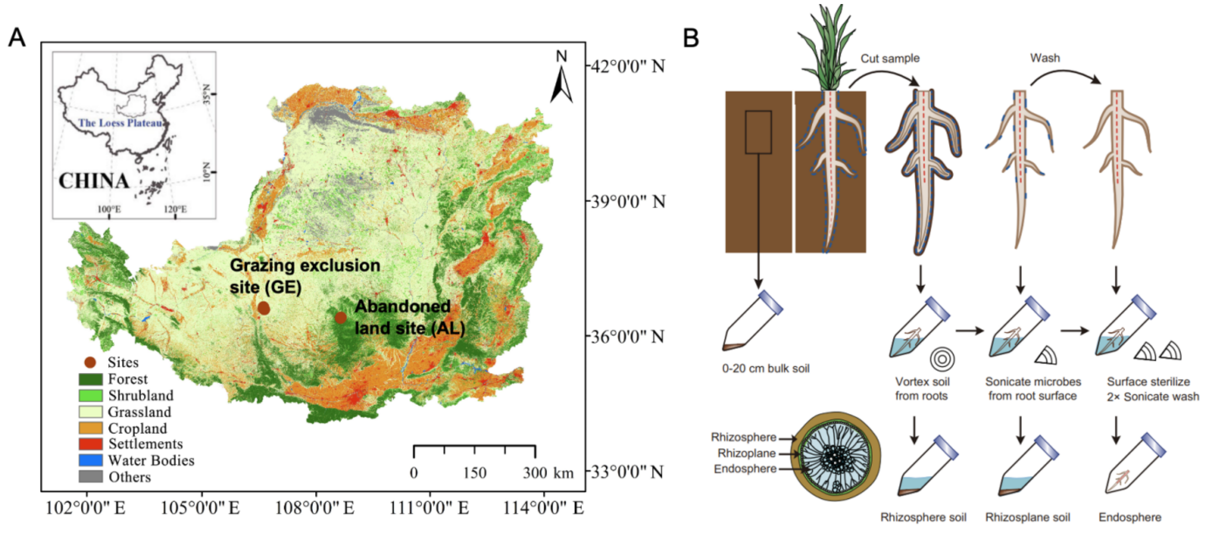

Figure S2 Microbial richness index (abundance-based coverage estimator, ACE) in each compartment of the main plant species in succession at (A) land abandonment site and (B) grazing exclusion site. Dots indicated means and error bars represent standard error. Lowercase letters indicate significant differences among succession year, which were performed by one-way ANOVA and Post hoc comparisons using Tukey's multiple-range test.

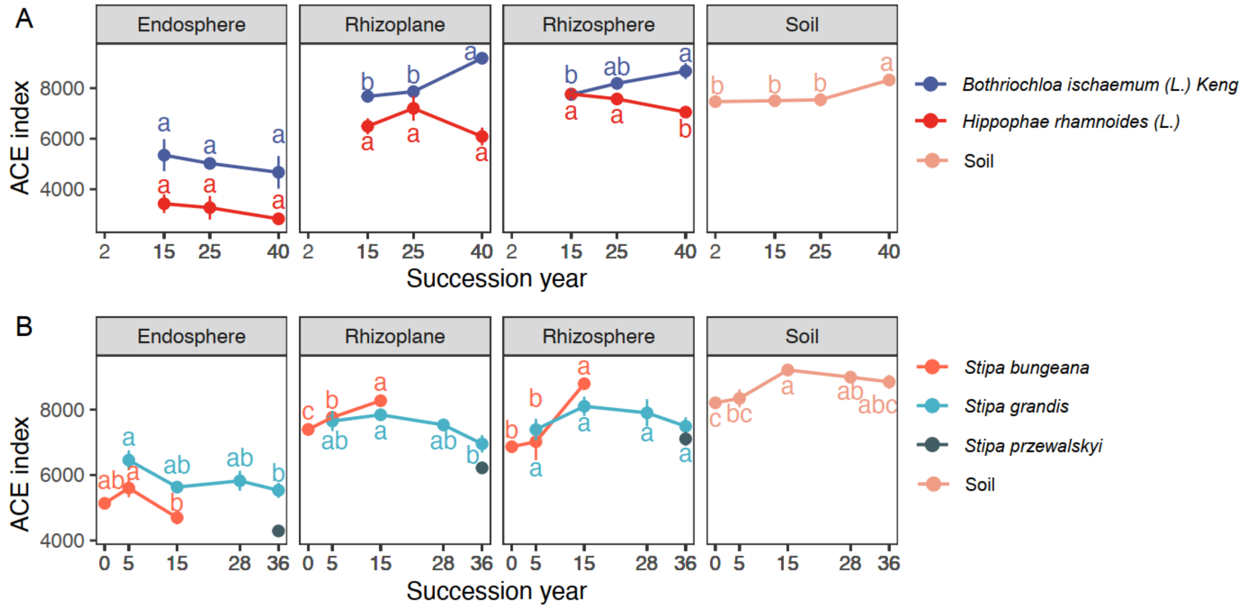

Figure S3 Relationships between the Shannon indices of soil and root-associated microbes and succession years at (A) land abandonment site and (B) grazing exclusion site. Linear regression were conduct between Shannon index and succession years in each compartment, R and P value were calculated.

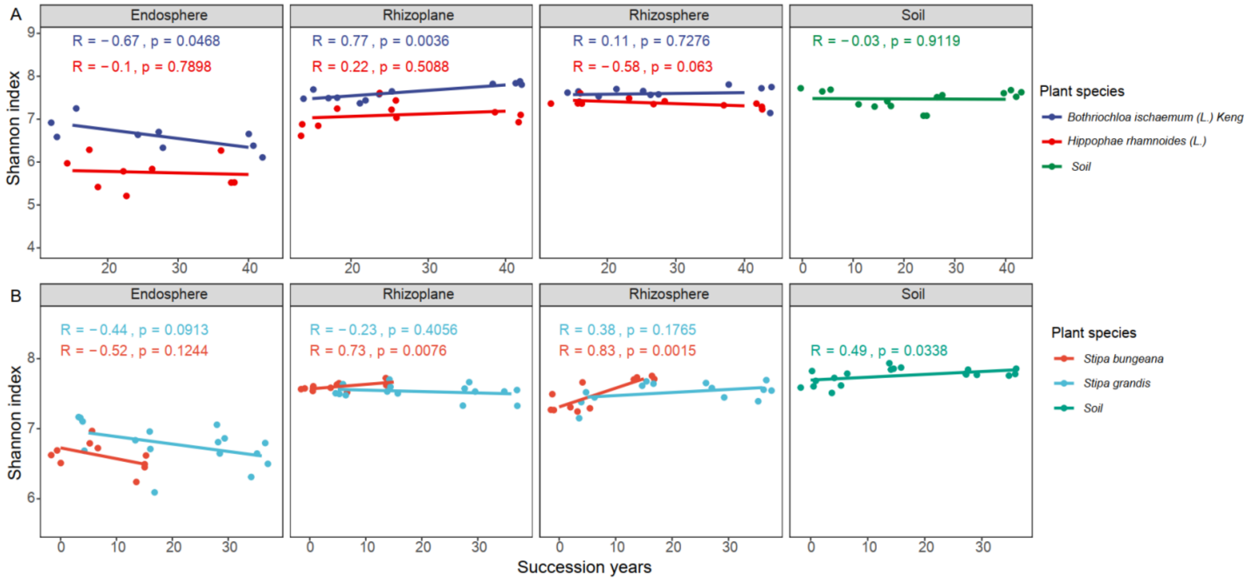

Figure S4 Relationships between the Shannon indices of soil and root-associated microbe and plant coverage along with succession at (A) land abandonment site and (B) grazing exclusion site. Linear regressions were conduct between Shannon index and plant coverage in each compartment, R and P value were calculated.

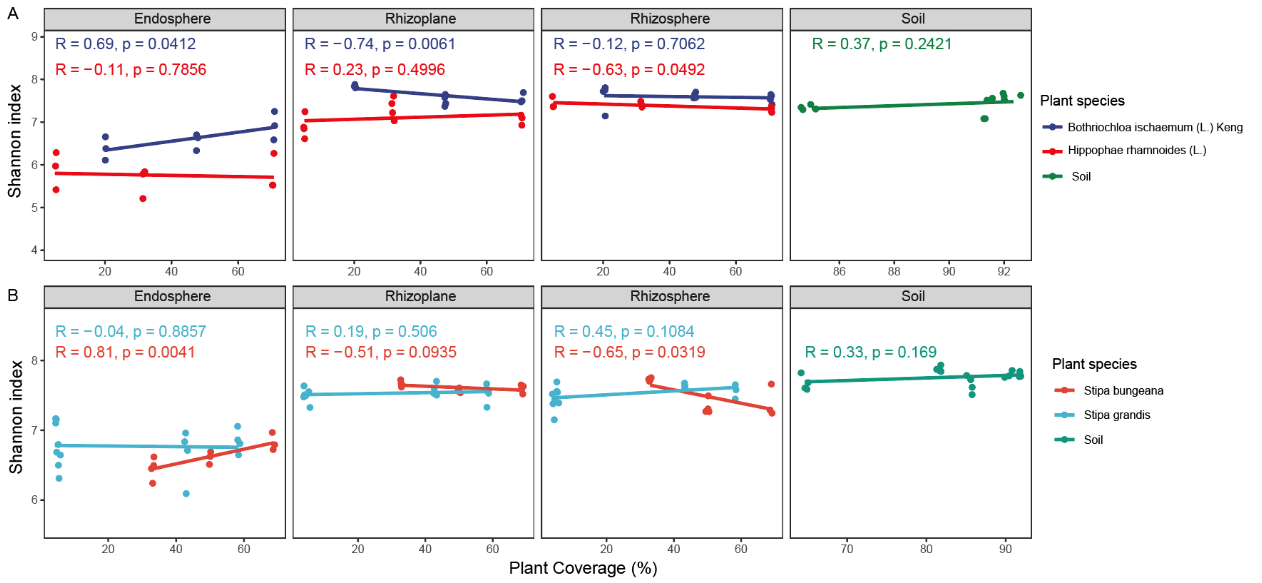

Figure S5 Changes in the soil and root-associated microbial communities of different plant species with succession at (A) land abandonment site and (B) grazing exclusion site. The similarity is calculated by 1- Bray–Curtis dissimilarity. Linear regression were conduct between similarity and succession time.

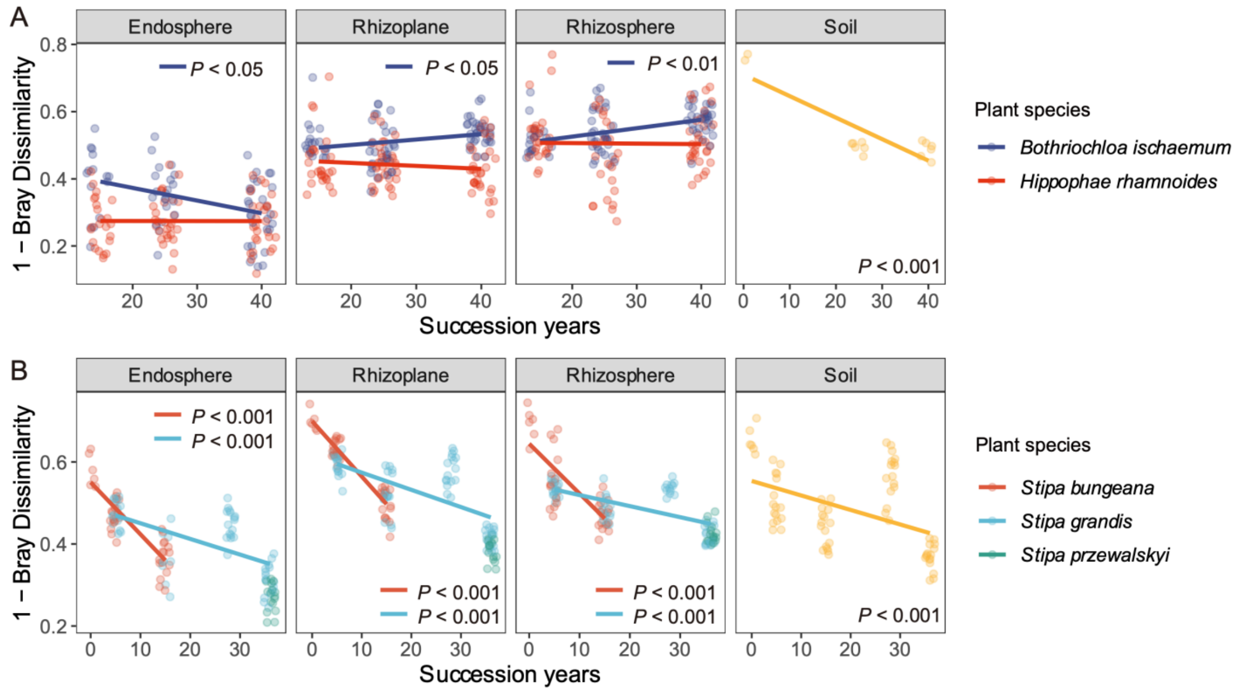

Figure S6 Microbial community similarity among soil and root-associated microbes during succession at (A) land abandonment site and (B) grazing exclusion site. The similarity is calculated by 1- Bray–Curtis dissimilarity. Linear regression were conduct between community similarity and succession years in each compartment.

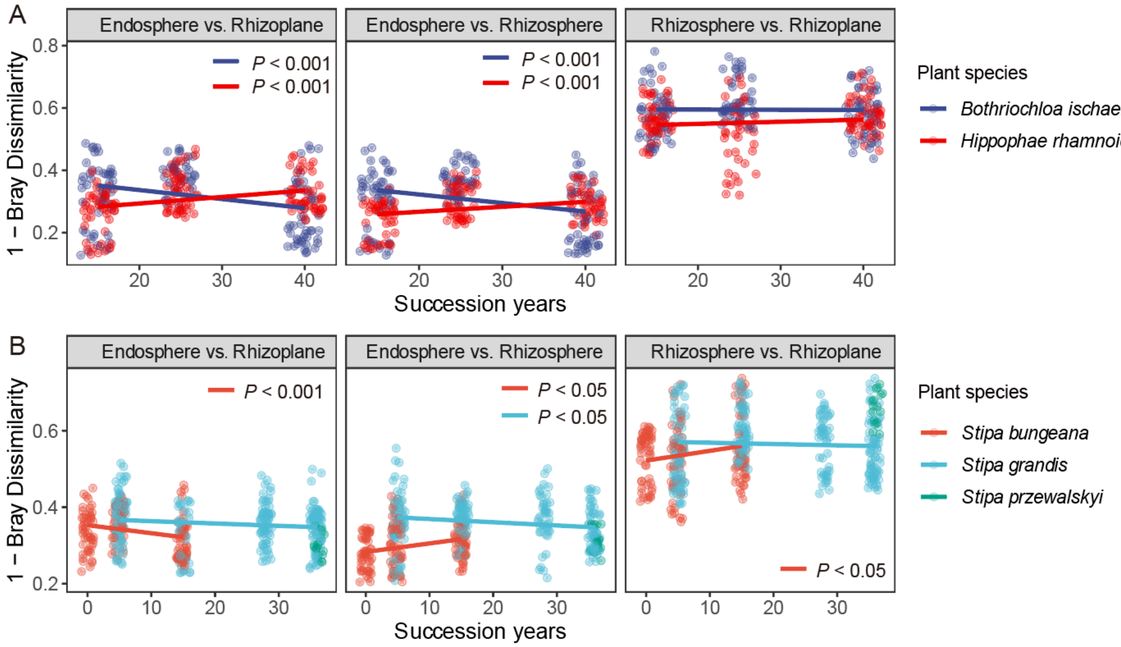

Figure S7 The relative abundance (left axis) and Shannon index (right axis) of common ASVs in the soil and root-associated microbes in dominant and nondominant plant species along with the two successional patterns. (A) is for is the pioneer species *Bothriochloa ischaemum* (L.) Keng at land abandonment site; (B) is for the late species *Hippophae rhamnoides* L. at land abandonment site. (C) is for the pioneer species *Stipa bungeana* at grazing exclusion site, and (D) is for the late species *Stipa grandis* at grazing exclusion site. Dots indicated means and error bars represent standard error. Lowercase letters indicate significant differences among succession year, which were performed by one-way ANOVA and Post hoc comparisons using Tukey's multiple-range test.

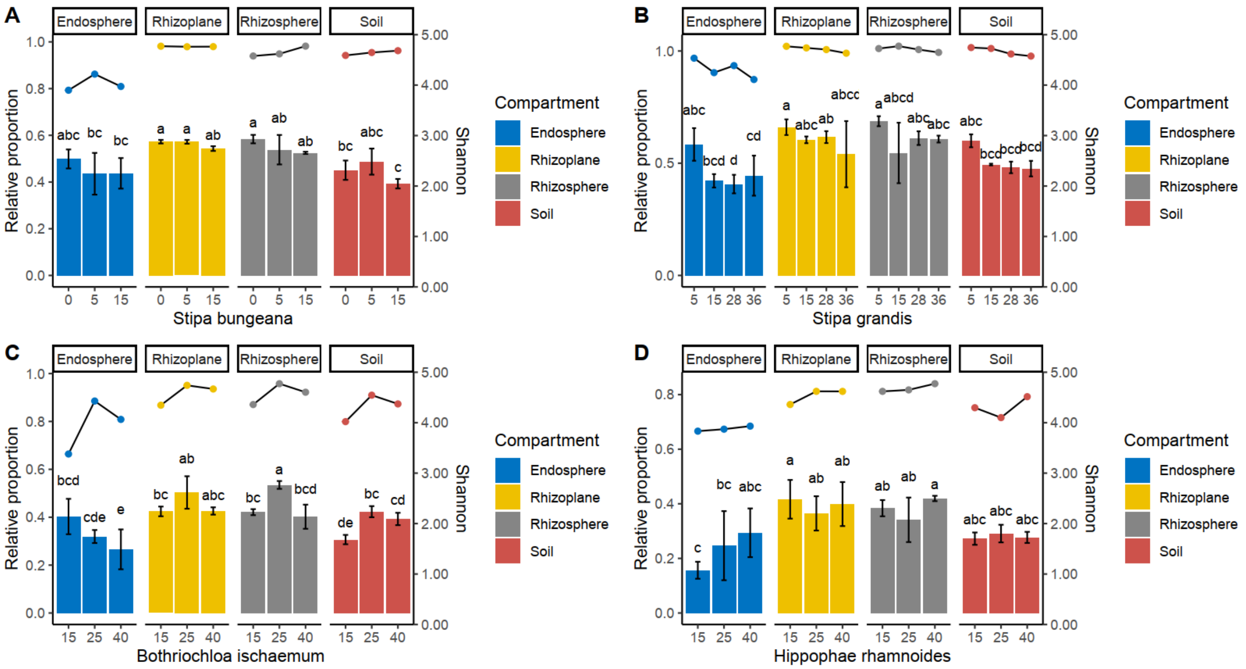

Figure S8 Estimates of the error rates for the number of selected ASVs via on full random forest (RF) models for dominant and nondominant plants along with succession at land abandonment site and grazing exclusion site.

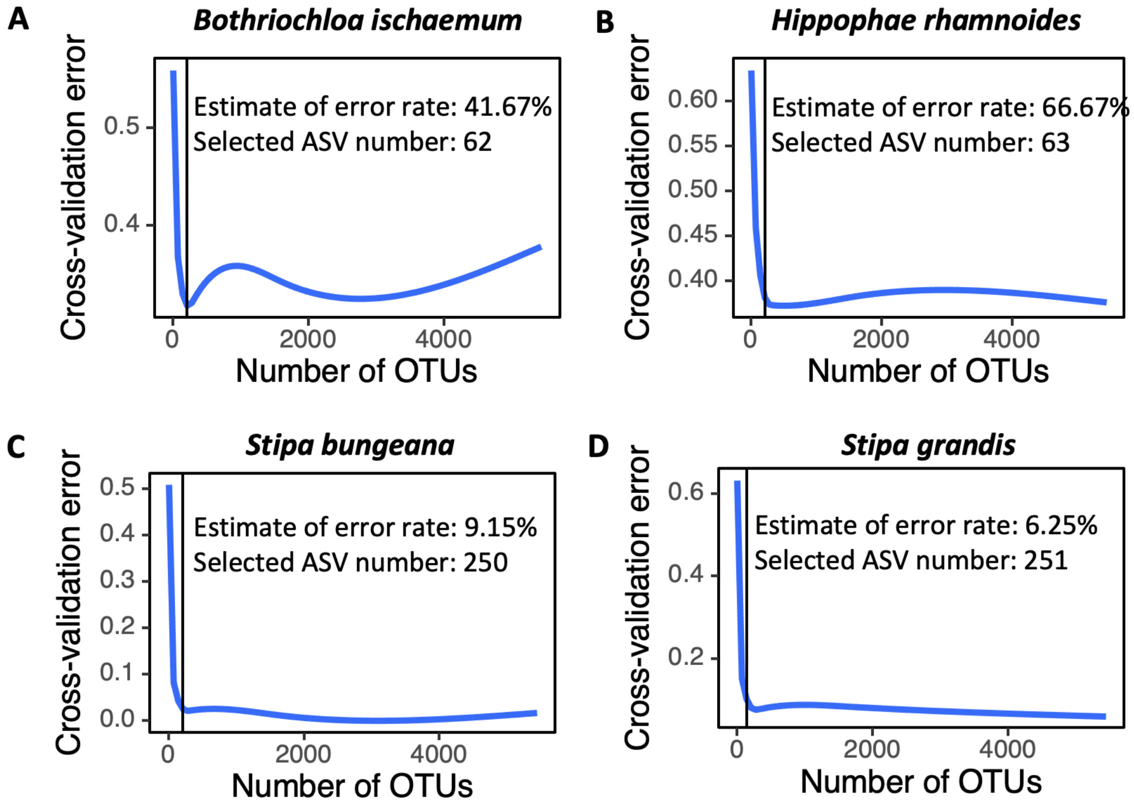

Figure S9 Abundance profiles of succession-discriminant ASVs from the endosphere via full random forest (RF) models for each plant species at land abandonment site. ASVs are colored by their classification as decreasing, increasing, or netural colonizers over the season, and these types of ASVs abundance in the surroundings (rhizoplane, rhizosphere and soil). Discriminant ASVs of the soil and root-associated microbe along with succession in abandoned land site. Neighbor joining tree of discriminant ASVs which showed significant changes in endosphere compared with the other compartments in (**A**) early successional *Bothriochloa ischaemum* and (**B**) late successional *Hippophae rhamnoides*. An outward blue, yellow and grey bars away from the tree represent early, late and complex colonizing of discriminant ASVs in endosphere, respectively. (**C**) Relative abundance of discriminant ASVs in soil-root continuum along with succession.

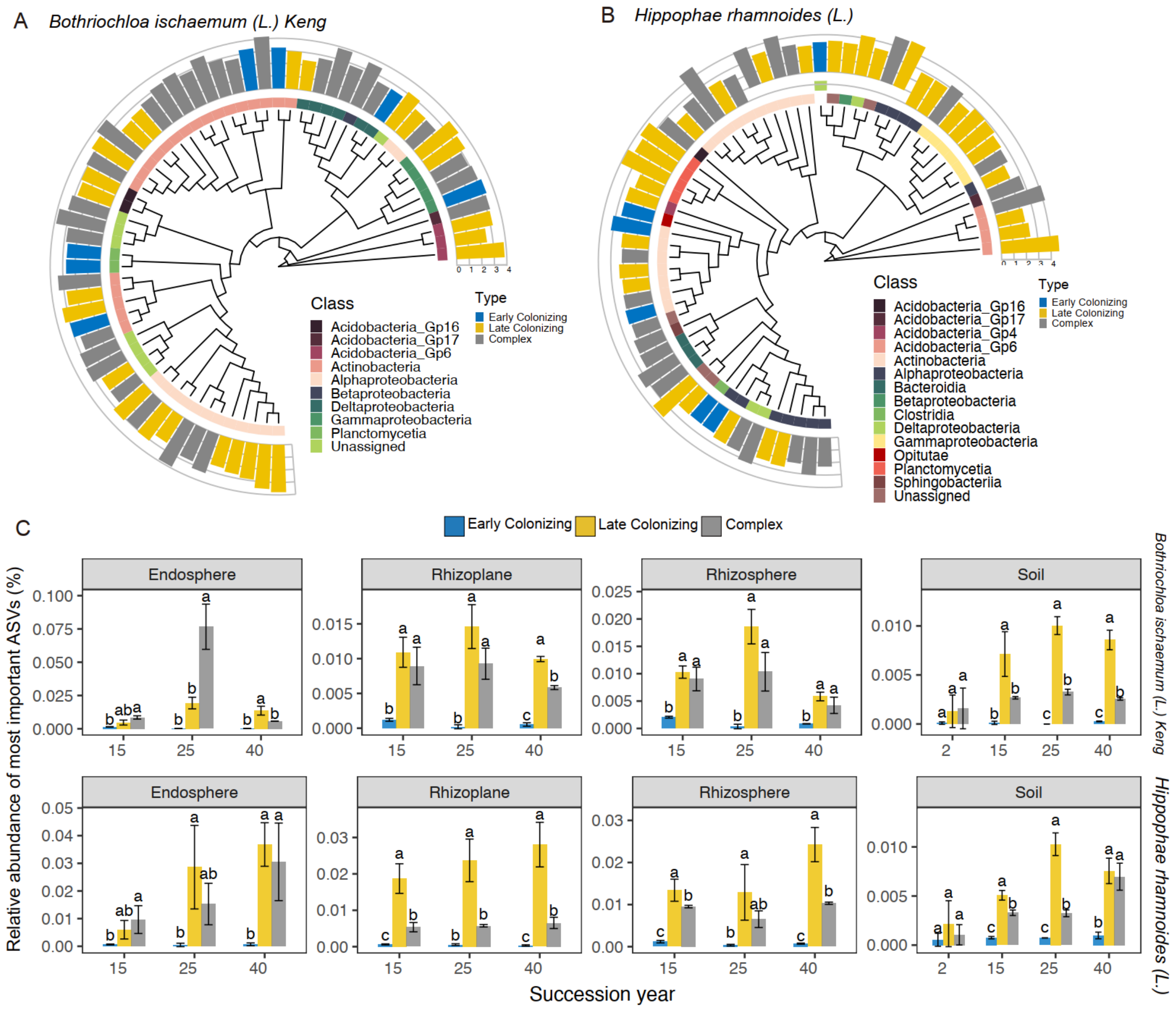

Table S1 Geographical characteristics, soil physicochemical properties, plant composition and study species coverage in each successional site and year.

Site	Succession year	Latitude (N)	Longitude (E)	SOC (g/Kg)	TN (g/Kg)	pH	Plant composition	Total coverage (%)	Study species coverage (%)		
									Stipa bungeana	*Stipa grandis*	*Stipa przewalskyi*
GE0	0	36°13′10″	106°23′3″	16.19±0.64	1.73±0.02	8.3±0.01	***Stipa bungeana*** *Potentilla acaulis* *Heteropappus altaicus*	64.7	50		
GE5	5	36°13′29″	106°23′30″	24.05±0.61	2.35±0.02	8.3±0.01	***Stipa bungeana*** *Anthoxanthum glabrum* ***Stipa grandis***	85.3	69	4.3	
GE15	15	36°11′56″	106°24′43″	20.5±0.74	2.00±0.01	8.09±0.03	***Stipa grandis*** ***Stipa bungeana*** *Thymus mongolicus*	81.7	33	43	
GE28	28	36°12′3″	106°24′40″	13.04±1.12	1.53±0.05	8.51±0.02	***Stipa grandis*** *Carex aridula* ***Stipa bungeana***	91.7	5.7	58.3	
GE36	36	36°15′05″	106°23′10″	28.94±0.90	3.04±0.03	8.35±0.03	***Stipa przewalskyi*** ***Stipa grandis*** *Thymus mongolicus*	90.3		5.7	72.3
									Bothriochloa ischaemum	*Hippophae rhamnoides*	
AL0	0	36°4′36″	108°31′21″	8.95±1.32	0.77±0.01	8.63±0.02	*Bothriochloa ischaemum*	1.3	1		
AL15	15	36°5′4″	108°31′37″	15.54±2.46	1.08±0.02	8.73±0.02	***Bothriochloa ischaemum, Hippophae rhamnoides,*** *Lespedeza bicolor*	85	71	5	
AL25	25	36°5′11″	108°31′32″	12.78±0.81	1.07±0.04	8.68±0.03	***Bothriochloa ischaemum, Hippophae rhamnoides*** *,Carex duriuscula*	91.3	48	31.7	
AL40	40	36°4′05″	108°31′01″	12.34±1.20	1.17±0.06	8.49±0.01	***Hippophae rhamnoides***, *Pyrus betulifolia Bge.* ***Bothriochloa ischaemum***	92.3	20	70.7	

Table S2 Permutational multivariate ANOVA (PERMANOVA) test of bacterial community based on the basis of Bray-Curtis distance matrix results.

	Abandoned land (AL)					Grazing exclusion (GE)				
	**Host plant**	**Years**	**Total reads**	**Chloroplast reads**	**Proportion of chloroplast reads (%)**	**Host plant**	**Years**	**Total reads**	**Chloroplast reads**	**Proportion of chloroplast reads (%)**
**Endosphere**	*Bothriochloa ischaemum (L.) Keng*	15	72352	9188	12.74±4.19	*Stipa bungeana (Trin)*	0	63580	1149	1.85±0.64
		25	54656	6184	12.08±6.51		5	67134	2856	4.24±2.83
		40	60934	7947	12.29±6.53		15	75131	5537	7.39± 3.90
	Hippophae rhamnoides (L.)					*Stipa grandis (P. G. Pallas)*	5	69795	4067	5.67±4.11
		15	63538	22602	35.50±11.37		15	72462	4126	5.84±4.41
		25	62676	20128	13.46±5.09		28	71930	10666	14.85±2.63
		40	53382	17279	28.89±12.24		36	70896	2945	7.36±6.65
						Stipa przewalskyi	36	67760	16606	24.75±2.30
**Rhizoplane**	*Bothriochloa ischaemum (L.) Keng*		75011	180	0.24±0.19	*Stipa bungeana (Trin)*		75377	40	0.05±0.01
	Hippophae rhamnoides (L.)		75551	4193	5.53±2.22	*Stipa grandis (P. G. Pallas)*		75969	60	0.08±0.05
						Stipa przewalskyi		74656	48	0.06±0.02
**Rhizosphere**	*Bothriochloa ischaemum (L.) Keng*		74678	90	0.12±0.05	*Stipa bungeana (Trin)*		75535	40	0.05± 0.02
	Hippophae rhamnoides (L.)		77290	1699	2.18±0.91	*Stipa grandis (P. G. Pallas)*		74393	67	0.09±0.04
						Stipa przewalskyi		75312	89	0.12 ±0.06
**Soil**			72447	16	0.02±0.01			73103	18	0.02±0.02
**Endosphere**			0.432	0.0701	<0.01**			0.366	**<0.001*****	**<0.001*****
**Rhizosphere**			0.086	**<0.001*****	**<0.001 *****			0.616	0.112	0.0937
**Rhizoplane**			0.402	**<0.001 *****	**<0.001 *****			0.566	0.385	0.406

Table S3 Permutational multivariate ANOVA (PERMANOVA) test of bacterial community based on the basis of Bray-Curtis distance matrix results.

	Df	SumsOfSqs	MeanSqs	F.Model	R^2	Pr(>F)	
Microhabitat	3	7.978	2.65925	22.0685	0.15496	0.001	***
Host plant	5	4.183	0.83653	6.9422	0.08125	0.001	***
Succession year	1	0.698	0.69842	5.796	0.01357	0.001	***
Succession pattern	1	0.855	0.85502	7.0957	0.01661	0.001	***
Microhabitat: Host plant	10	11.475	1.1475	9.5228	0.22289	0.001	***
Microhabitat: Succession year	3	1.087	0.36239	3.0074	0.02112	0.001	***
Host plant: Succession year	3	1.099	0.36646	3.0412	0.02135	0.001	***
Succession year: Succession pattern	1	0.264	0.26351	2.1868	0.00512	0.013	*
Microhabitat: Host plant: Succession year	6	1.912	0.31869	2.6447	0.03714	0.001	***

Table S4 Permutational multivariate ANOVA (PERMANOVA) of the bacterial communities based on the basis of Bray-Curtis distance at each successional site.

	Abandon Land (AL)					Grazing exclusion (GE)				
	Df	SumOfSqs	F	Pr(>F)		Df	SumOfSqs	F	Pr(>F)	
Microhabitat	3	0.021793	10.8303	**0.001**	***	3	0.0065159	23.9141	**0.001**	***
Succession year	1	0.001433	2.1365	0.062	.	1	0.0012986	14.2982	**0.001**	***
Host plant	1	0.002743	4.0899	**0.003**	**	**3**	0.0013073	4.7979	**0.001**	***
Microhabitat: Succession year	3	0.002143	1.065	0.35		3	0.0009399	3.4496	0.001	***
Microhabitat: Host plant	2	0.001555	1.1589	0.294		6	0.0010502	1.9271	0.001	***
Succession year: Host plant	1	0.001056	1.5739	0.163		1	0.0006254	6.8863	0.001	***
Microhabitat: Succession year: Host plant	2	0.001087	0.8102	0.566		2	0.000411	2.2624	0.003	**
Residual	71	0.047623				105	0.0095365			

Table S5 Mantel test between microbial community and soil physicochemical factors of each microhabitat at each succession site.

		Abandon Land (AL)		Grazing exclusion (GE)	
		R^2	*P*	R^2	*P*
Endosphere	SOC	-0.050	0.707	0.104	0.080
	TN	0.027	0.391	**0.183**	**0.009**
	TP	-0.157	0.968	-0.032	0.628
	NH4+	-0.141	0.904	**0.177**	**0.029**
	NO3-	0.034	0.367	0.111	0.095
	pH	**0.149**	**0.043**	0.082	0.152
	All	-0.111	0.815	**0.134**	**0.037**
Rhizosphere	SOC	-0.074	0.839	**0.484**	**0.000**
	TN	0.011	0.408	**0.606**	**0.000**
	P	0.106	0.113	0.110	0.078
	NH4+	-0.027	0.565	**0.165**	**0.025**
	NO3-	0.018	0.398	0.177	0.012
	pH	0.060	0.191	0.000	0.465
	All	0.035	0.346	**0.272**	**0.001**
Rhizoplane	SOC	0.045	0.281	**0.362**	**0.000**
	TN	-0.076	0.684	**0.405**	**0.000**
	TP	0.042	0.316	0.145	0.050
	NH4+	0.093	0.210	**0.319**	**0.001**
	NO3-	-0.145	0.875	**0.234**	**0.004**
	pH	-0.062	0.748	-0.004	0.502
	All	-0.003	0.495	**0.391**	**0.000**
Soil	SOC	**0.612**	**0.001**	**0.476**	**0.000**
	TN	**0.675**	**0.000**	**0.512**	**0.000**
	TP	**0.691**	**0.000**	-0.012	0.491
	NH4+	-0.125	0.760	-0.011	0.479
	NO3-	**0.292**	**0.038**	**0.180**	**0.040**
	pH	0.243	0.062	-0.050	0.667
	All	**0.626**	**0.001**	**0.189**	**0.023**
